# Implementing Spatial Segregation Measures in R

**DOI:** 10.1371/journal.pone.0113767

**Published:** 2014-11-21

**Authors:** Seong-Yun Hong, David O'Sullivan, Yukio Sadahiro

**Affiliations:** 1 Center for Spatial Information Science, University of Tokyo, Tokyo, Japan; 2 Department of Geography, University of California, Berkeley, California, United States of America; Fondazione Edmund Mach, Research and Innovation Centre, Italy

## Abstract

Reliable and accurate estimation of residential segregation between population groups is important for understanding the extent of social cohesion and integration in our society. Although there have been considerable methodological advances in the measurement of segregation over the last several decades, the recently developed measures have not been widely used in the literature, in part due to their complex calculation. To address this problem, we have implemented several newly proposed segregation indices in R, an open source software environment for statistical computing and graphics, as a package called **seg**. Although there are already a few standalone applications and add-on packages that provide access to similar methods, our implementation has a number of advantages over the existing tools. First, our implementation is flexible in the sense that it provides detailed control over the calculation process with a wide range of input parameters. Most of the parameters have carefully chosen defaults, which perform acceptably in many situations, so less experienced users can also use the implemented functions without too much difficulty. Second, there is no need to export results to other software programs for further analysis. We provide coercion methods that enable the transformation of our output classes into general R classes, so the user can use thousands of standard and modern statistical techniques, which are already available in R, for the post-processing of the results. Third, our implementation does not require commercial software to operate, so it is accessible to a wider group of people.

## Introduction

The measurement of segregation has been a topic of debate and discussion among sociologists and geographers for decades [1]–[6]. Many measures have been proposed over the last half century, to capture various dimensions of this complex social phenomenon, but only a few of them have been regularly used in the segregation literature. Some of the indices have not been adopted in practice because they overlap with the existing ones to a large extent, providing little new insight into the patterns of segregation [3], and some have not been chosen due to their methodological flaws and ambiguity in interpretation [1], [4].

There are, however, a number of good methods that have theoretical advantages over the conventional ones but have been rarely used, primarily due to the computational difficulties. Recently developed spatial indices might be cases in point: the calculation procedure of these measures tends to be more sophisticated than the traditional counterparts, and it often involves spatial data processing using Geographic Information Systems (GIS) techniques [7]. Although considerable efforts have been made in recent years to implement these spatial indices [7]–[9], most examples either do not incorporate important improvements in the field, or they require commercial software to run, which is not available to the public.

To address this problem, we have developed an R package **seg** that provides facilities for theoretically compelling spatial segregation measures. R is a multi-platform, open-source software environment for statistical computing and graphics, so it is accessible to almost all members of the academic and research communities. Furthermore, since R offers numerous powerful statistical and graphical tools, the manipulation and visualisation of the spatial data, as well as the post-processing of the results can be readily performed without exporting it to another data format.

This paper describes the structure and key features of the **seg** package, with as little technical details as possible. A complete explanation of usage, syntax, arguments, and code examples is given elsewhere, such as in the Help documentation that is distributed with the program. In the next section, we present the definitions of the segregation measures currently implemented and explain briefly how they work. The subsequent section evaluates the reliability and computational efficiency of the implemented functions with a set of hypothetical segregation patterns: the idealised landscapes are adopted from Morrill [10] and Wong [11], as they are intended to test the accuracy of the associated functions through regression testing. This paper concludes with a discussion about the limitations of the current work and future directions for development.

## Methods

The measures of segregation can be classified based on a number of different criteria. Massey and Denton, for instance, examined 20 indices available at that time and grouped them into five categories, based on their correlations to each other [3]. The indices may also be distinguished into spatial and non-spatial indices, depending on whether the calculation is sensitive to the spatial arrangement of the population. One well-known example of the latter is the index of dissimilarity developed by Duncan and Duncan [12], while a considerable number of more recent methods, such as the set of measures proposed by Reardon and O'Sullivan [13], belong to the former.

In this paper, we classify the segregation measures under two headings, namely, zone-based and surface-based measures, based on the types of input data required. This classification follows the one in Kramer et al. [2]. Zone-based measures use aggregated population counts for their calculation, and surface-based measures utilise a continuous population density surface to minimise the so-called modifiable areal unit problem (MAUP) [14]. We distinguish segregation measures in this manner because the amount of information required for the calculation significantly differs between the two, and hence the computational steps are also very different. Table 1 presents the zone-based and surface-based segregation measures implemented in the **seg** package; each of these will be discussed in the following subsections.

**Table 1:** A list of segregation measures currently implemented in the seg package.

Measure	Surface-based?	Spatial?	Function
Index of dissimilarity, 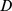 [12]	No	No	dissim()
Modified 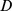 (contiguity), 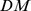 [10]	No	Yes	dissim()
Modified 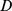 (boundary length), 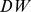 [11]	No	Yes	dissim()
Modified 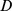 (perimeter/area ratio), 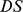 [11]	No	Yes	dissim()
Index of spatial proximity, 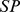 [15]	No	Yes	isp()
Concentration profile [16]	No	No	conprof()
Spatial exposure/isolation index, 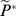 [13]	Yes	Yes	spseg()
Spatial information theory index, 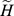 [13]	Yes	Yes	spseg()
Spatial relative diversity index, 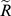 [13]	Yes	Yes	spseg()
Spatial dissimilarity index, 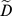 [13]	Yes	Yes	spseg()
Decomposable measure of segregation, 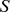 [18]	Yes	Yes	deseg()

The mathematical definitions of 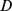 and 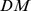 are given in this paper. For the other measures, see the corresponding papers cited in the table.

### Zone-based measures

The calculation of the zone-based measures is relatively straightforward: most can be calculated by hand, or using a simple spreadsheet program. There are, however, several more complex methods that demand extensive data preparation. The **seg** package provides tools for some of these methods, including the index of dissimilarity [12] and its spatially-modified forms [10], [11], the index of spatial proximity [15], and the concentration profile [16]. In this section, we present a brief introduction to these indices and their implementation in R. More detailed descriptions of the methods are given in the corresponding original papers.

The index of dissimilarity, 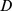, is one of the most widely used measures in the segregation literature. For the study region consisting of 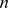 census tracts, 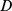 is defined as: 
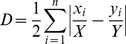
(1)where 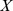 and 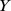 denote the total population counts of two population groups, and 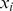 and 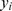 are the local populations in the census tract 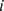.

Although 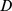 itself is non-spatial, this can be adjusted to reflect the spatial distribution of the population. For example, Morrill [10] suggested adding a spatial term to (1), so it becomes: 
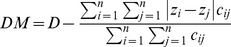
(2)where 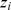 and 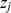 are the proportions of the minority population in the census tracts 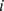 and 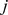, respectively, and 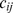 denotes an element at 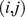 in a contiguity matrix **C**, which becomes one only if 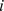 and 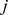 are adjacent.

This spatially-adjusted version of 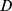 can be further supplemented by taking into account additional geometric features of the spatial units, which might influence individuals' accessibility to neighbouring areas. Wong [11] proposed two alternative indices, 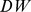 and 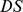. 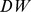 uses the lengths of shared borders between census tracts as a weighting factor that replaces the binary contiguity matrix in (2). *DS* incorporates the perimeter and area of census tracts into the measurement.

These three spatial associates of 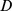 might be more realistic representation of residential segregation, but they—particularly, 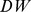 and 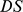
—are rather complicated to calculate. To facilitate the use of these extended indices, we have implemented them in the **seg** package, as a single function called dissim(). One function is sufficient for all these measures, because the calculation procedures of 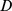, 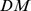, 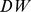 and 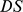 are essentially identical: the difference lies in the amount of spatial information required, not in the way in which they are calculated.

Table 2 shows a list of supported input and output classes for dissim(), along with the other functions implemented in the package. The input for dissim() can be a spatial object, such as a shapefile imported into R, or an 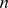-by-2 table, where 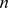 is the number of census tracts and the two columns contain the population counts of mutually exclusive groups. While the aspatial version of the index is always calculated regardless of the type of the input, the other three indices are computed only when the input is a spatial object and required libraries are already installed on the user's machine.

**Table 2:** Supported input and output classes for the implemented functions.

Function	Supported input classes	Output class
dissim()	SpatialPolygons, SpatialPolygonsDataFrame, matrix, data.frame	list
isp()	SpatialPoints, SpatialPointsDataFrame, SpatialPolygons, SpatialPolygonsDataFrame, matrix, data.frame	vector
conprof()	matrix, data.frame	list
spseg()	SpatialPoints, SpatialPointsDataFrame, SpatialPolygons, SpatialPolygonsDataFrame, matrix, data.frame	SegSpatial
deseg()	SpatialPoints, SpatialPointsDataFrame, SpatialPolygons, SpatialPolygonsDataFrame, matrix, data.frame	SegDecomp

Each function can take an object of any R class in *Supported input classes* and return an object of *Output class* upon successful execution. Note that SegSpatial and SegDecomp are custom defined S4 classes.

The spatial adjustment is not made when the input is a data frame, unless an optional spatial weight matrix, whose elements describe the social and physical distances between census tracts, is provided by the user. If the weight matrix is a simple binary matrix indicating the adjacency between the units, the adjusted value is equivalent to 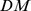. If, on the other hand, it is a numeric matrix representing the standardised lengths of common boundaries, or the perimeter-to-area ratio, the function returns 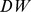 or 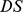, respectively.

The output ranges from 0 and 1, where a value of zero represents no segregation and a value of one indicates complete segregation. Theoretically, the spatially-adjusted indices are similar to the traditional version when the census tracts with a high proportion of the minority population are clustered together in the study region (i.e., positive spatial autocorrelation). If areas with similar population composition are dispersed, however, 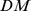, 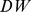 and 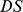 should be considerably lower than that from (1), as the additional spatial component becomes large.

It is noteworthy that the index of dissimilarity and its spatial associates consider only two population groups at a time. Considering that many societies are increasingly diverse in terms of race, ethnicity, culture, and religion, this limitation is not desirable. One of the classical, zone-based measures that can work with multiple groups is the index of spatial proximity 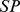 [15], which compares the average distance between members of the same population group with that between different groups. In general, 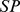 is calculated based on an assumption that all people in each census tract is located at a representative point, such as the centroid of the tract, and thus, its reliability is limited by the validity of this assumption.

In the **seg** package, the function to compute this measure of clustering is called isp(). The input for this function must be a spatial object, whether it is a SpatialPolygons object or a matrix with coordinates (Table 2), because 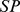 does not have an aspatial analog. Another difference between dissim() and isp() is that the data table (i.e., population counts) for isp() does not have to be a matrix with only two columns; it accepts a numeric matrix with more than two columns, as 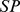 can handle multiple population groups. By default, a simple negative exponential function is used to control how the distance affects the social interactions between people, but different distance decay models can be specified through an optional argument if necessary.

The function returns a single numeric value indicating the degree of segregation: a value of one means absence of segregation, and values greater than one indicate clustering. If the index value is less than one, it indicates an unusual form of segregation (i.e., people live closer to other population groups).

It is important to note that although the index of spatial proximity is a useful method for evaluating the degree of residential clustering in the study region, it tends to neglect geographic patterns of small minorities by definition. If one's interest lies in identifying residential clustering of an individual population group, the concentration profile approach proposed by Poulsen et al. [16] might be more suitable.

A concentration profile is a cumulative distribution curve, whose vertical and horizontal values represent the proportion of the subject group and their share in census tracts, respectively. If, for example, the curve goes through the point (0.3, 0.4), it means that 40% of this population group reside in the areas where they comprise at least 30% of the local demographic composition. While this is conceptually similar to the Lorenz curve, in the segregation literature the Lorenz curve is often constructed by plotting the cumulative proportion of one population group against that of the other group. The concentration profile is different from the Lorenz curve in the sense that it plots the cumulative proportion of the population group against their relative demographic share in spatial units [17].

Concentration profiles can be produced using a function called conprof(). Unlike the other functions implemented in the **seg** package, conprof() does not require any spatial information as this approach is non-spatial (Table 2). Upon successful run, the function draws a concentration profile on the current graphic device and returns a numeric value ranging between 0 and 1. The return value is a summary statistic for the concentration profile, 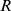, which is derived as described in Hong and Sadahiro [17]. This output can be interpreted in a similar manner to the index of dissimilarity: a small value indicates that the group comprises similar proportions of the local population in all census tracts, and a large value implies a high degree of residential concentration.

### Surface-based measures

The segregation measures in the previous subsection work on the data in which the population counts are agglomerated into arbitrarily defined geographic areas, such as census tracts, electorates, and school zones. Therefore, the resulting degree of segregation depends not only on the actual distribution of the population but also on the choice of spatial units [14].

Unlike the zone-based measures outlined above, the surface-based measures do not require the use of aggregate spatial units, so they are theoretically free from this problem. Although, in practice, almost all data available are provided in aggregate form, a plausible population density surface can be obtained using a variety of interpolation techniques by making certain assumptions regarding the distribution of the population [13]. This sort of approach does not necessarily eliminate all possible errors, but previous studies argued that it could reveal important patterns that would not be found using the conventional index of dissimilarity [2].

Nonetheless, the surface-based measures have not been as widely used in the literature as they might deserve to be. There has been hesitation among scholars to employ these indices, partly because their calculation is complicated and constructing an appropriate interpolation map requires significant computing skills and knowledge in statistics. In order to lower such computational barriers and facilitate the use of these potentially useful methods, we have implemented two sets of surface-based measures in the **seg** package. One is the spatial segregation indices developed by Reardon and O'Sullivan [13], which consists of the general spatial exposure/isolation index (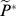), the spatial information theory index (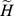), the spatial relative diversity index (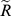), and the spatial dissimilarity index (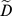). The other is the decomposable segregation measure proposed by Sadahiro and Hong [18].

The core function for the former method is called spseg(). As with isp(), the spseg() function requires a spatial object as an input. Ideally, this is a point data set that describes the residential locations of individuals, but aggregate spatial data can also be used. If the input is a polygon feature class, the function converts it to a population density surface by making one of the following assumptions:

1. All the population is located at the centroid of the census tract (default).

2. The population is uniformly distributed within each census tract.

3. The kernel density estimator (KDE) can approximate closely the true distribution of the population. If this option is chosen, spatial interpolation is performed through the kernel2d() function in the **splancs** package.

By default, the interpolation process is performed on a 100-by-100 grid. However, this spatial resolution of the output surface can, and should, be changed so that each cell in the output is sufficiently smaller than the original spatial units.

In the case where none of the above assumptions are considered acceptable, the user may want to employ other interpolation techniques, such as kriging, dasymetric mapping or pycnophylactic interpolation [19]. Although the **seg** does not provide facilities for these methods, there is already a wide range of user-contributed packages available in R (e.g., **geoR** and **gstat** for kriging/cokriging). The raster output from other R packages can also be used for spseg(), once it is coerced to a point class.

In the current implementation, the relationship between physical distance and social interaction is described by either an exponential function (i.e., 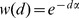) or a power function (i.e., 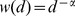), where 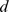 is the distance between two units and 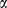 is the decay rate specified by the user. One can control the scale at which segregation is measured by changing these decay parameters (e.g., the larger the decay rate, the smaller the scale). If a maximum value for 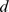 is given, any spatial units that are further than the specified distance will not be considered while evaluating the local demographic mix. As will be demonstrated in the next section, the use of this option can help enhance the computation speed, with little or no practical impact on the output.

**Figure 1:**
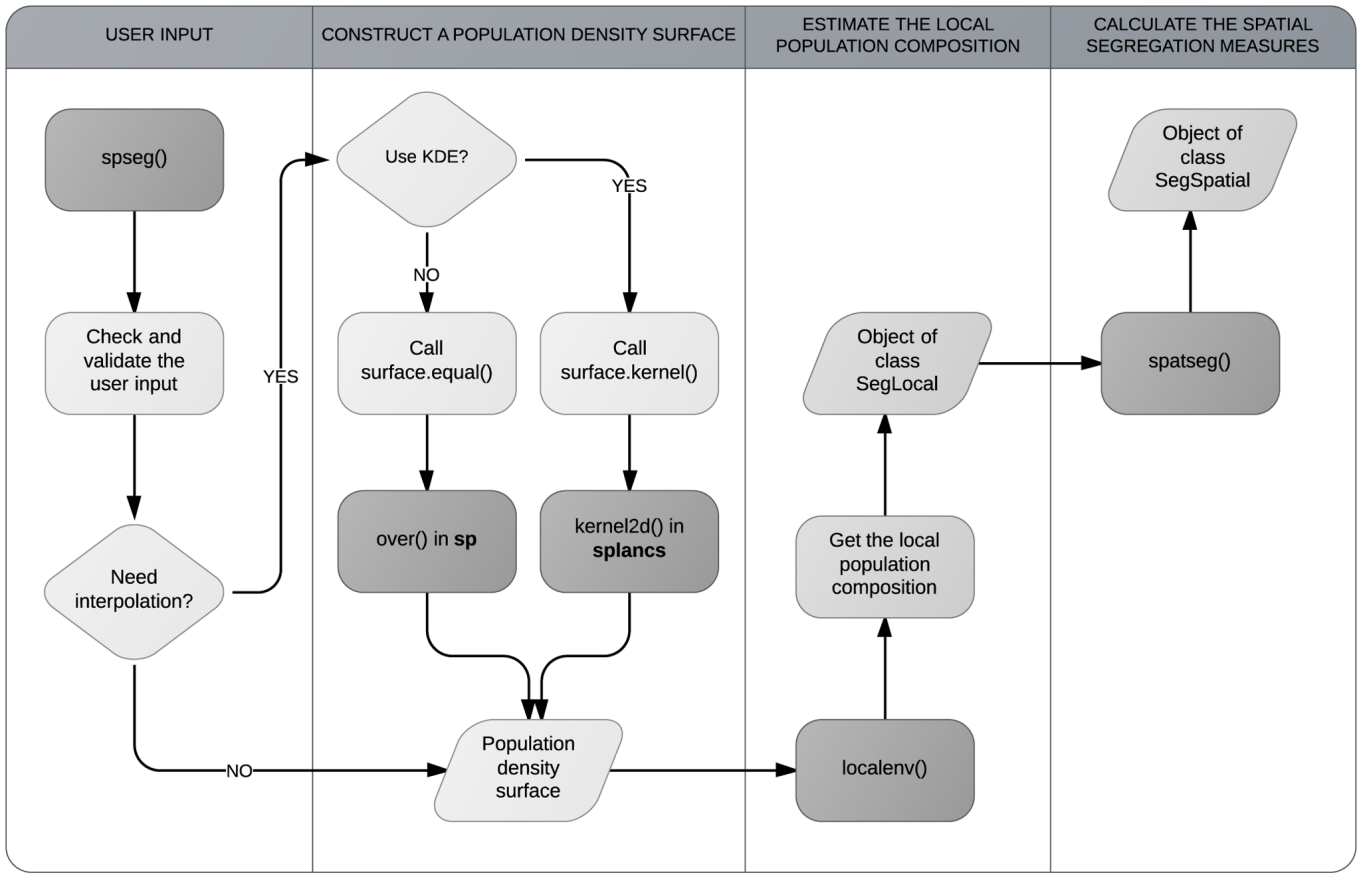
Computational flow of the spseg() function. It calls a series of subfunctions to calculate the spatial segregation measures. In this diagram, the curved-rectangles represent R functions and processes, the parallelograms refer to R objects, and the diamonds indicate the user options. Among the rectangles, only the shaded ones are user-level functions.

Once the function is called with appropriate options, it invokes a series of subroutines to accomplish the calculation (Figure 1). Although these subroutines are designed to work in sequence within the main function, they can also be run on their own. This modularisation is particularly advantageous when one wants to repeat only part of the calculation procedure. For instance, suppose that the user is interested in how the level of segregation changes with scale. One way to test this is multiple calls to spseg() with different scale arguments while holding other arguments constant. This is, however, computationally redundant, because it leads to the construction of the same population density surface each time it runs. It would be more efficient if we execute the subroutines separately, as it allows repeating the necessary components only.

In addition, it offers more flexibility in terms of data preparation. As mentioned above, spseg() employs a negative exponential distance decay function to model the effect of the distance on social interactions. While this simple function has been commonly adopted in the literature and is considered appropriate for general use [15], more realistic representation of neighbourhood may yield a more reliable estimate of segregation. If the user has irregularly-shaped neighbourhood boundaries generated from other R extension packages, or from other software, the local demographic composition could be manually calculated and passed to spatseg() directly, instead of going through all the steps in Figure 1.

**Figure 2:**
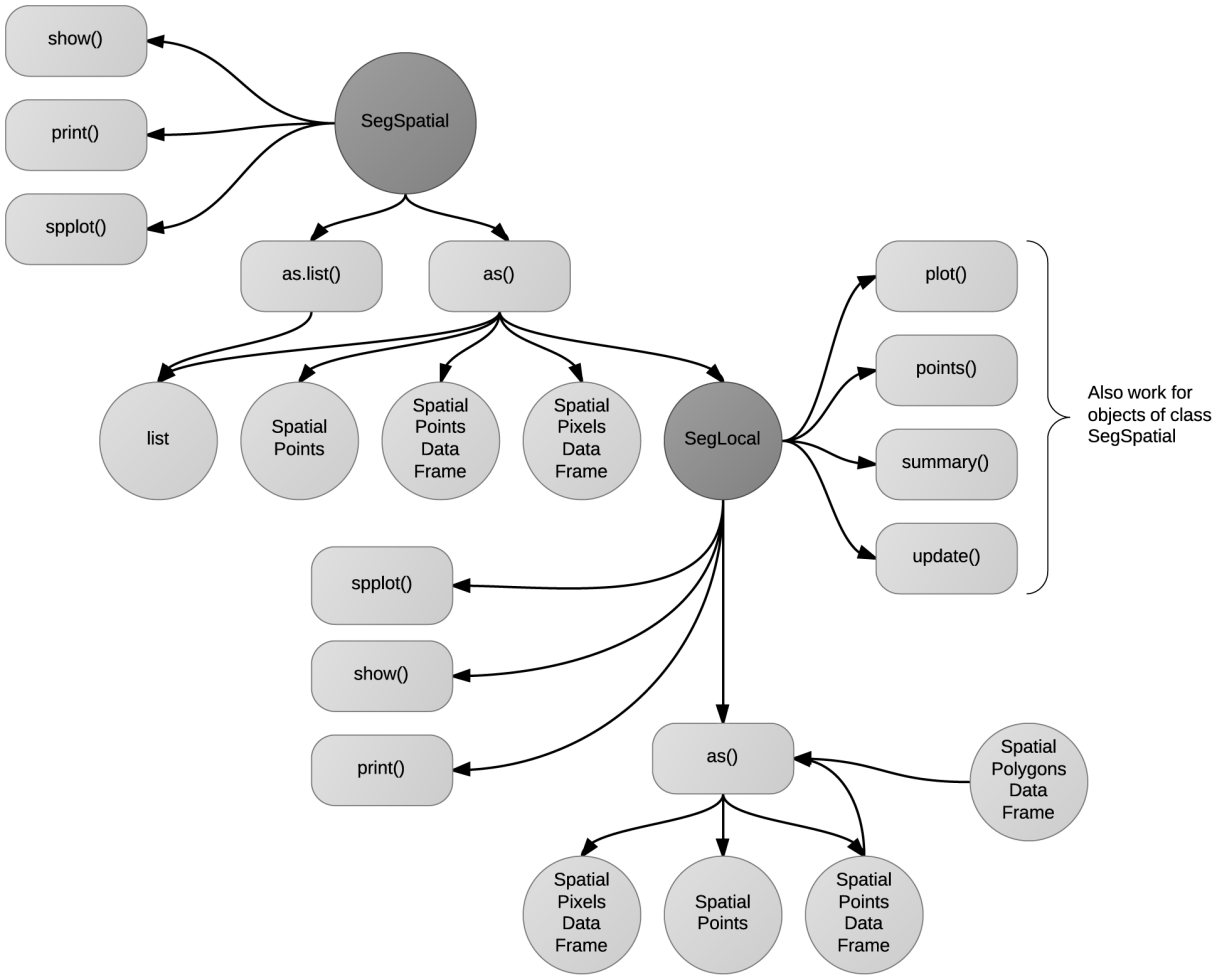
Available methods for SegSpatial and SegLocal. SegSpatial is a S4 class that stores results from the spseg() function. It inherits from another S4 class SegLocal.

Regardless of whether the final subroutine spatseg() is invoked from the main function spseg() or by a direct call, it always returns an object of class SegSpatial. This is a custom defined class and is designed to hold not only the calculated segregation indices but also information about the input spatial data. In order to access, retrieve and visualise the values in this class, we provide methods for some standard generic functions, including show(), print(), and plot(). Figure 2 displays a list of the generic functions that have a method for SegSpatial.

Another surface-based approach implemented in the **seg** package is the decomposable segregation measure, 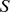 [18]. One advantage of this method is that it allows decomposing the estimated level of segregation into three independent components, namely, locational segregation, compositional segregation, and qualitative segregation. By evaluating each of these components separately, one can identify whether the observed segregation is mainly due to the demographic structure in the study region, such as the number of ethnic groups and their sizes, or it is caused by geographic clustering/isolation of certain groups.

In the **seg** package, there exists a function called deseg() to calculate this decomposable measure of segregation. It works in much the same way as spseg(): most of the computation steps are identical to those for spseg(), except that it does not utilise a distance decay function during the measurement of segregation. This method assumes that the input point data have been interpolated using KDE before the calculation, so the impact of distance on social interactions does not need to be modelled again.

**Figure 3:**
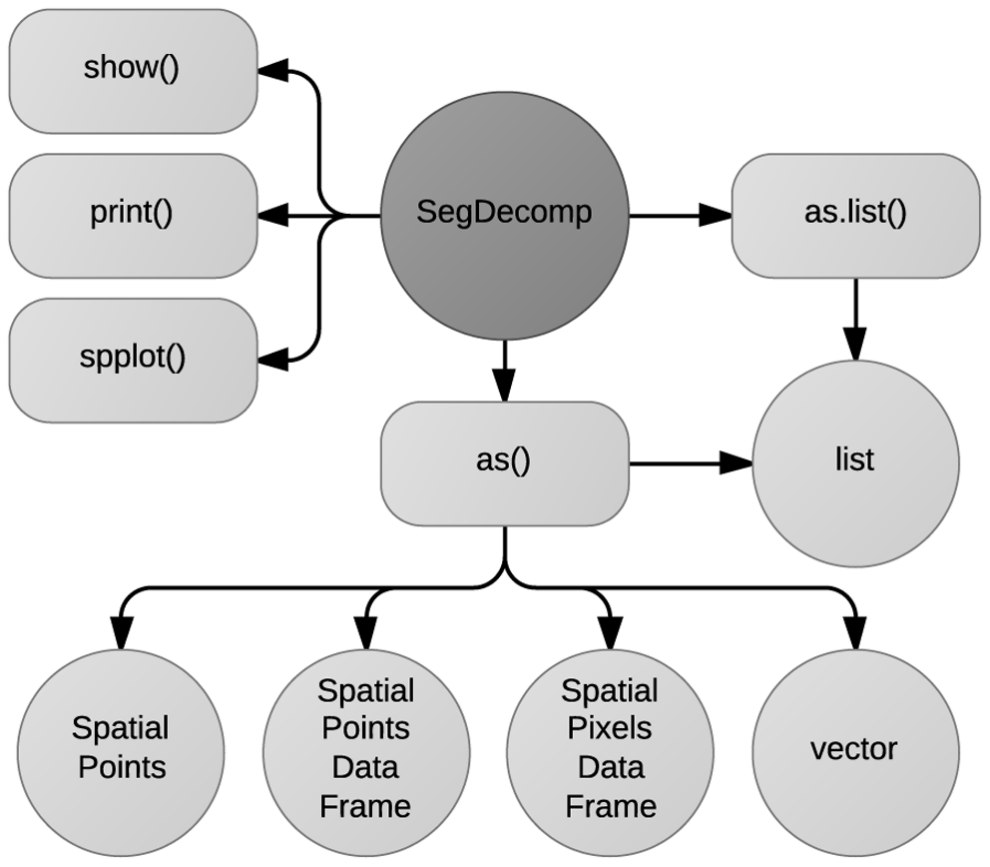
Available methods for SegDecomp. SegDecomp is a custom defined S4 class, containing the measured segregation from deseg().

Upon successful run, deseg() returns an object of class SegDecomp, which is another custom defined class in the **seg** package. The structure of this class is essentially the same as that of SegSpatial, and it can be manipulated and plotted using the methods implemented in the package (Figure 3).

## Results

### Accuracy

The **seg** package has a sample data set of eight different distributions of the population for demonstration and maintenance purposes. The data set itself is a simple data frame but can be displayed on a 10-by-10 grid to reproduce the hypothetical segregation patterns used by Morrill [10] and Wong [11]. Although these hypothetical patterns are not presented in this paper due to copyright restrictions, Table 3 provides references to the relevant figures in the original citation.

**Table 3:** A list of hypothetical segregation patterns adopted in this paper and their original citation.

Pattern	Morrill [10]	Wong [11]
A	p. 28, Figure 1 (1)	p. 562, Figure 1 (1)
B	p. 28, Figure 1 (2)	p. 562, Figure 1 (2)
C	p. 28, Figure 1 (3)	p. 562, Figure 1 (3)
D	p. 28, Figure 1 (4)	na
E	p. 28, Figure 1 (5)	na
F	na	p. 562, Figure 1 (4)
G	na	p. 562, Figure 1 (5)
H	na	p. 562, Figure 1 (6)
I	na	p. 568, Figure 6 (1)
J	na	p. 568, Figure 6 (2)
K	na	p. 568, Figure 6 (4)
L	na	p. 568, Figure 6 (5)
M	na	p. 568, Figure 6 (6)

For convenience, the patterns are named alphabetically in the order of their appearance in the literature. ‘na’ indicates that the pattern does not appear in the corresponding paper.

Example code in the package documentation applies the implemented functions to this sample data set, with various combinations of user input, not only to illustrate their use but also to ensure that they produce the *expected* output. Since the same spatial configurations have been used in the previous studies [10], [11], we can easily determine whether the results from dissim() are correct or not by comparing them with the values in those earlier works.

Table 4 presents the output from the dissim() function for these synthetic landscapes, hereafter referred to as patterns A-M as described in Table 3. Although some of the results seem to be slightly different from the measurements of Morrill [10] (i.e., 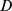 for the pattern D and 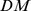 for the patterns C and D), it is consistent with the one in Wong [11] for the pattern C. Considering that the differences between dissim() and Morrill [10] are relatively minor, this might be due to rounding errors.

**Table 4:** 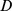
, 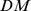, 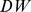 and 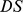 for the hypothetical segregation patterns listed in Table 3.

	dissim()				Morrill [10]		Wong [11]			
	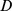	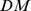	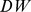	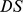	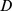	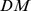	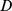	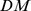	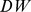	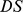
A	1.00	0.94	0.94	0.95	1.00	0.94	1.00	0.94	0.94	0.95
B	1.00	0.83	0.83	0.84	1.00	0.83	1.00	0.83	0.83	0.84
C	1.00	0.50	0.50	0.54	1.00	0.48	1.00	0.50	0.50	0.54
D	0.84	0.79	0.79	0.79	0.83	0.76	na	na	na	na
E	0.83	0.66	0.66	0.68	0.83	0.66	na	na	na	na
F	1.00	0.97	0.97	0.97	na	na	1.00	0.97	0.97	0.97
G	1.00	0.93	0.93	0.93	na	na	1.00	0.93	0.93	0.93
H	1.00	0.90	0.90	0.91	na	na	1.00	0.90	0.90	0.91
I	1.00	0.50	0.50	0.50	na	na	1.00	0.50	0.50	0.50
J	1.00	0.33	0.24	0.54	na	na	1.00	0.33	0.24	0.54
K	1.00	0.50	0.70	0.74	na	na	1.00	0.50	0.70	0.74
L	1.00	0.57	0.54	0.68	na	na	1.00	0.50	0.54	0.68
M	1.00	0.40	0.36	0.61	na	na	1.00	0.33	0.36	0.57

‘na’ indicates that the value is not available in that paper. The results are rounded to the second decimal place.

**Figure 4:**
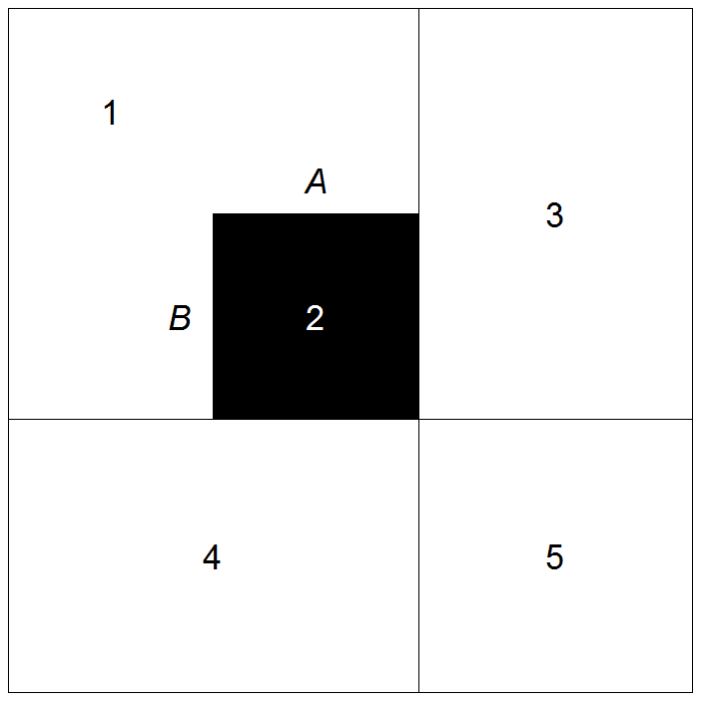
A sample pattern of segregation. The white and black cells are where the minority population comprises 0% and 100% of the local population, respectively. The numbers inside of the cells indicate the cell ID, and the letters denote the edges.

In comparison, the differences in 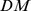 for the last two patterns are fairly large, and it is perhaps because dissim() uses a different way of counting the neighbouring pairs from Wong [11]. For example, dissim() identifies seven pairs of neighbours from Figure 4: 1–2, 1–3, 1–4, 2–3, 2–4, 3–5, and 4–5. In four of these neighbouring pairs, the spatial units have the same population composition (i.e., 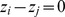 in the equation (2)). The remaining three pairs consist of one unit with, say, Asians only, and the other unit with non-Asians only. This makes 
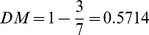
. Wong [11], however, distinguished the neighbouring pairs by the edge, so there would be eight pairs of neighbours, not seven: 1–2 through the edge A, 1–2 through the edge B, 1–3, 1–4, 2–3, 2–4, 3–5, and 4–5. As a result, the spatial component in (2) changes to 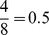, and 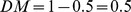.

Another notable difference appears in 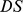 for the pattern M. The cause for this discrepancy is not certain, but one possible explanation is that the lengths of shared borders between census tracts were rounded to the first decimal place during the calculation of 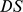 in Wong [11]. If we calculate the index in this way, dissim() generates the same result as Wong [11], up to the second decimal place.

Table 5 displays 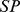, 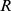 and the surface-based spatial segregation measures for the same data set. Unfortunately, there is no control output (i.e., results from a known set of the data) available for these indices, so the quality of the results cannot be assessed in the same manner as the index of dissimilarity. Nonetheless, the changes in the measured segregation with respect to the changes in the patterns seem to suggest that these functions also produce plausible results.

**Table 5:** 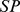
, 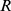 and the surface-based spatial segregation measures for the hypothetical segregation patterns listed in Table 3.

	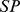	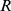	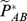	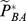	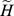	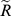	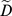	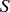
A	1.62	1.00	0.18	0.06	0.68	0.71	0.83	0.94
B	1.16	1.00	0.49	0.16	0.23	0.26	0.48	0.82
C	0.89	1.00	0.75	0.25	0.00	0.00	0.03	0.63
D	1.44	0.67	0.35	0.11	0.50	0.51	0.71	0.85
E	1.12	0.66	0.58	0.18	0.16	0.17	0.40	0.76
F	1.42	1.00	0.30	0.03	0.66	0.61	0.86	0.96
G	1.34	1.00	0.51	0.06	0.40	0.32	0.69	0.93
H	1.16	1.00	0.64	0.07	0.33	0.23	0.67	0.90
I	0.67	1.00	0.09	0.03	0.78	0.82	0.90	0.98
J	0.26	1.00	0.63	0.18	0.13	0.16	0.32	0.76
K	0.91	1.00	0.17	0.01	0.79	0.79	0.89	0.99
L	0.37	1.00	0.49	0.09	0.41	0.39	0.67	0.88
M	0.34	1.00	0.50	0.09	0.42	0.38	0.68	0.87

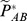
 is the exposure of the minority group (i.e., the black cells in the figures) to the majority group (i.e., the white cells), and 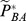 is that of the majority group to the minority group. All calculations were done with default settings, except that KDE was used for the spatial segregation measures. The results are rounded to the second decimal place.

### Computation speed

**Figure 5:**
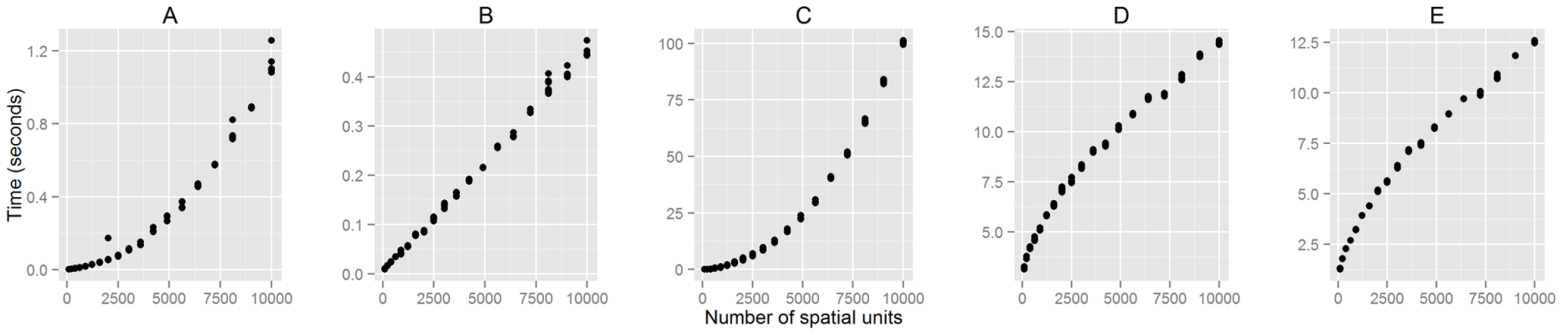
Computation time of the implemented functions for different input size. As the number of spatial units increases, the computation time also increases for all functions but at a different rate. The functions tested here are: dissim() (A), conprof() (B), isp() (C), spseg() (D), and deseg() (E).

All tests in this subsection were performed on a computer running OS X 10.9.4 and R 3.1.0 with a 1.70 GHz Intel Core i7 processor and 8 GB of RAM. Figure 5A–5B shows that all the zone-based tools perform quickly: when applied to a 10-by-10 grid, the dissim() function with an optional spatial weight matrix completed the calculation in less than 0.03 seconds from 20 iterations, and conprof() took only around 0.01 seconds on the average. As the size of the data increased, the amount of time required to obtain results also increased, but not to a large extent. The computation speed does not seem to be too much of an issue here.

In the case of isp(), on the other hand, an increase in the number of spatial units tends to slow down the process rapidly: it ran in less than 0.03 seconds for a simple 10-by-10 grid, but this figure grew up to about 92.35 seconds for a larger, 100-by-100 grid, as it involves the construction and manipulation of a 10,000-by-10,000 matrix (Figure 5C). This is not very slow, but a caution is probably needed when applied to a larger data set, because the current implementation always uses spaces proportional to 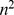, where 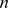 is the number of spatial units.

The computation speed of the surface-based measures is also influenced by the number of spatial units (Figure 5D–5E), as it affects the construction of the population density surface. However, for spseg() and deseg(), there are more important factors that impact on the computation times, such as the number of measurement points, the kernel bandwidth (if KDE is used), and the distance decay parameters. Ideally, the number of measurement points should be as many as possible for an accurate estimation of segregation, because the level of segregation an individual experiences changes continuously over space. The kernel bandwidth should be chosen to ensure that the estimated density surface provides a plausible representation of the actual distribution of the population, and the distance decay parameters should reflect the intensity of social interactions between locations.

**Figure 6:**
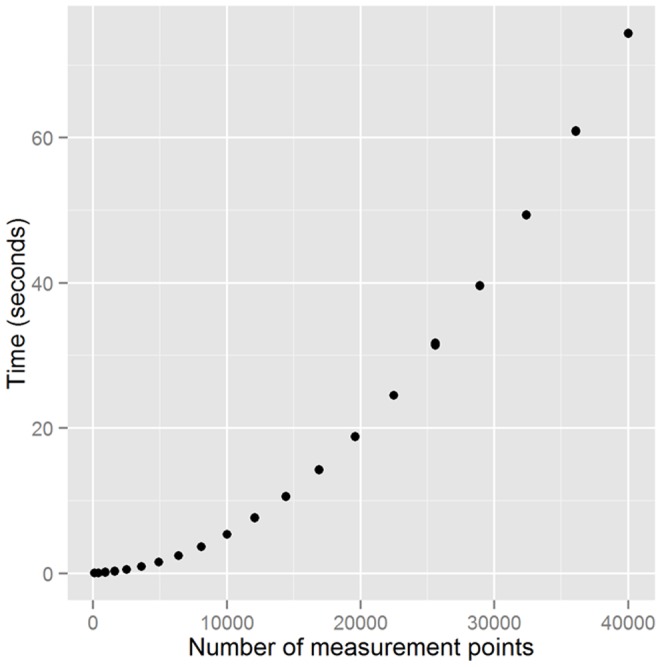
Relationship between the computation time and the number of measurement points for spseg().

In real word applications, however, the large number of measurement points compared to the spatial resolution of input data often slows down the calculation significantly, while making little difference in the output. Figure 6 shows that as the dimensions of the grid increase, the computation time also increases at an exponential rate for the same data set. Nonetheless, the changes in the spatial information theory index, 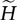, from the spseg() function seem to be negligible: when a 10-by-10 grid (i.e., 100 measurement points in total) was superimposed on the pattern A, it took only around 0.02 seconds to complete the task, and 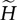 was 0.697. This value remained quite similar (i.e., 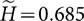), even when a much larger, 200-by-200 grid was employed, but the computation time increased up to 74.4 seconds on the average.

This result is of course data-dependent, and sometimes a fine grid is desired to produce more accurate estimates of segregation. In this case, a maximum distance for the distance decay function can be specified to improve the running speed: for example, when spseg() was run on the pattern A with default values, it spent more than 5 seconds to return 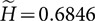. However, when an optional maximum distance was provided to the function, the computation time was reduced by more than one third (i.e., 3.501 seconds), and almost the same figure (i.e., 0.6853) was obtained. In general, the smaller this value, the faster the calculation, but it should be large enough to make sure that 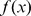 is practically zero, where 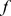 is the distance decay function and 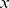 is the maximum distance chosen, to minimise its impact on the output.

In the case of the kernel bandwidth, there is lots of literature on a data-driven choice of this value, but it is often useful to examine several candidates first, as it could shed some light on the scale of segregation. In a similar vein, although a simple negative exponential function with a decay factor of 1 or 2 has been conventionally used as the distance decay parameters since White [15], the use of varying distance decay rates can help reveal the scale of segregation present in the study region. Reardon et al. [9], for example, demonstrated how changes in the distance decay parameters affect measured segregation using segregation profiles. This implies that unlike the number of measurement points, the kernel bandwidth and the distance decay parameters should be chosen more carefully, not on the basis of computational considerations.

## Discussion

In this paper, we have described our implementation of segregation measures in R. The **seg** package contains a number of useful zone-based and surface-based measures of segregation, and among these, the concentration profile approach and the decomposable segregation measure are not available elsewhere. Although there are a few recently developed standalone applications and add-on packages that provide access to 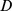 and its spatial associates [7], [8], and the spatial segregation measures [9], the present implementation has a number of advantages over the existing tools.

First, our implementation is flexible in the sense that it provides detailed control over the calculation process with a wide range of input parameters. Most of the parameters have carefully chosen defaults, which perform acceptably in many situations, so less experienced users can also use the implemented functions without too much difficulty.

Second, there is no need to export the results to other software programs for further analysis. Since the **seg** package works within the R environment, thousands of standard and modern statistical techniques, as well as facilities for data manipulation and visualisation, can be used to analyse and map the results. This is an important advantage, especially over the standalone applications, because the measurement of segregation is often the beginning of research, not the end. Once the presence of segregation is identified, the next step is to investigate its cause and potential consequences, and a variety of exploratory and confirmatory methods in R can be very useful in this phase. To help the use of various other extension packages in R, we provide coercion methods that enable the transformation of our output classes into more general R classes.

Third, the **seg** package does not require commercial software to operate, so it is accessible to a wider group of people. R is an open-source software program, and the **seg** package is downloadable from the Comprehensive R Archive Network (CRAN) without charge. Considering the high cost of commercial statistical and GIS software, our implementation in R can be a more cost effective alternative for researchers and students.

It should be noted that the **seg** package aims to provide implementations of the measures that have been developed specifically for residential segregation research. While more general approaches, such as the G statistics [20] and the local Moran statistic [21], may also be utilised as an indicator of residential clustering [22]–[24], they are already available elsewhere, for example, in the **GeoXP** and **spdep** packages.

At present, our implementation is limited to several place-based segregation measures that assess the demographic diversity of certain geographic areas. Although there are many other, potentially important segregation measures that are not currently covered by the package, most of them are relatively simple and do not require extensive computing power. In this work, we focused on providing tools for those that are more complex and demand more advanced skills and resources. In future work, we will try to incorporate some of the classical indices, as well as newly developed activity space-based approaches [25], [26], into the **seg** package.
